# Supplementary material for: Complex‐centric proteome profiling by SEC‐SWATH‐MS
Source: Mol Syst Biol. 2019 Jan 14;15(1):e8438. doi: 10.15252/msb.20188438 (PMC6346213; doi:10.15252/msb.20188438)
Supplement: Supplementary file 7 — Dataset EV6 [file MSB-15-e8438-s007.zip › feature_plots_bioplex/Q9BT49.pdf]

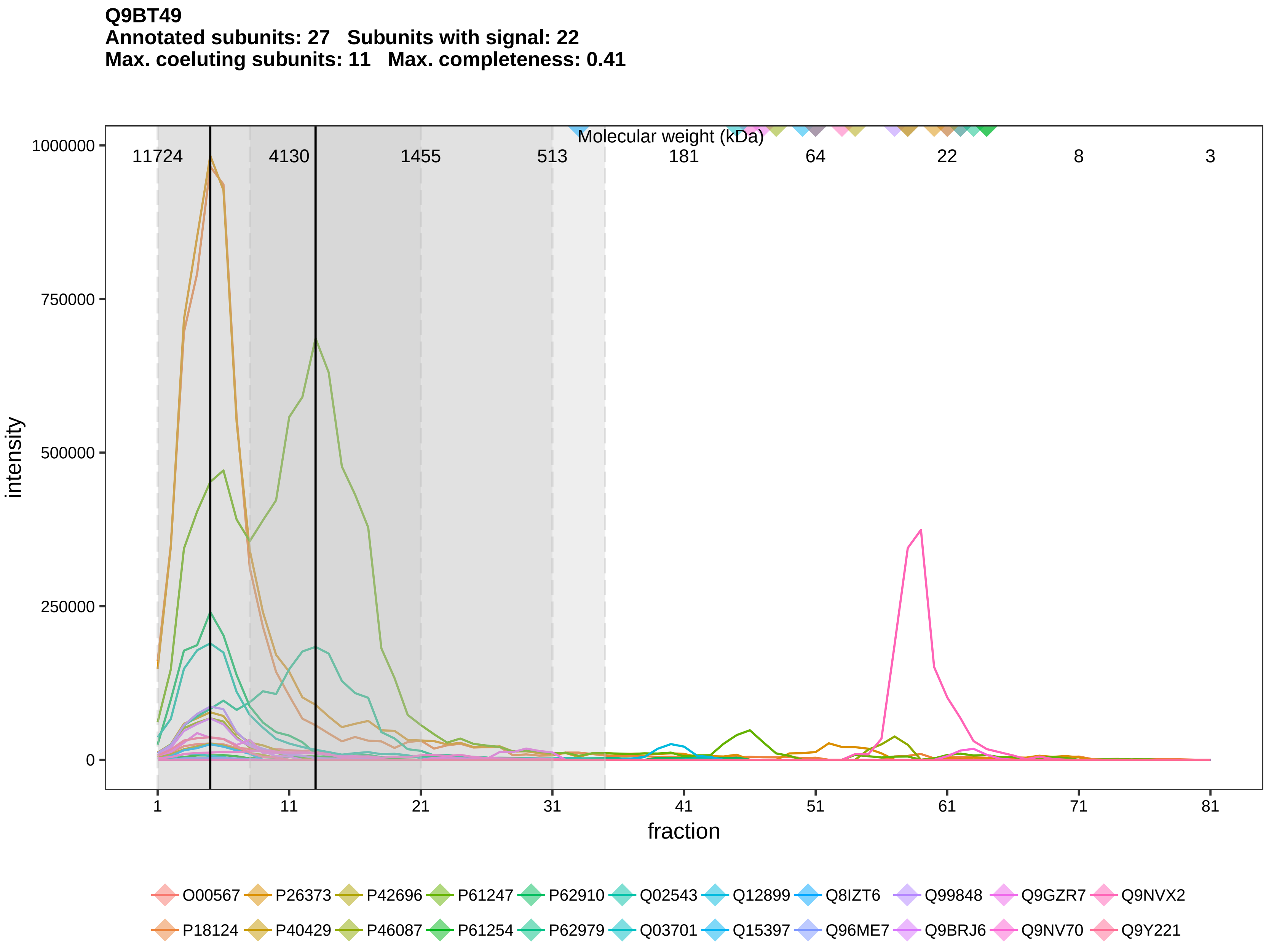

Q9BT49
Annotated subunits: 27 Subunits with signal: 22
Max. coeluting subunits: 11 Max. completeness: 0.41
Molecular weight (kDa)
11724
4130
1455
513
181
64
22
8
3
1000000
750000
500000
250000
0
intensity
1
11
21
31
41
51
61
71
81
fraction
O00567
P26373
P42696
P61247
P62910
Q02543
Q12899
Q8IZT6
Q99848
Q9GZR7
Q9NVX2
P18124
P40429
P46087
P61254
P62979
Q03701
Q15397
Q96ME7
Q9BRJ6
Q9NV70
Q9Y221
